# Modeling the Restricted Mean Survival Time Using Pseudo‐Value Random Forests

**DOI:** 10.1002/sim.70031

**Published:** 2025-02-22

**Authors:** Alina Schenk, Vanessa Basten, Matthias Schmid

**Affiliations:** ^1^ Institute for Medical Biometry, Informatics and Epidemiology, Medical Faculty University of Bonn Bonn Germany; ^2^ Department of Mathematics, Informatics and Technology Koblenz University of Applied Sciences, Rhein‐Ahr‐Campus Remagen Germany

**Keywords:** breast cancer survival, pseudo‐values, random forest, restricted mean survival time, survival analysis, treatment contrast

## Abstract

The restricted mean survival time (RMST) has become a popular measure to summarize event times in longitudinal studies. Defined as the area under the survival function up to a time horizon τ>0, the RMST can be interpreted as the life expectancy within the time interval [0,τ]. In addition to its straightforward interpretation, the RMST allows for the definition of valid estimands for the causal analysis of treatment contrasts in medical studies. In this work, we introduce a non‐parametric approach to model the RMST conditional on a set of baseline variables (including, e.g., treatment variables and confounders). Our method is based on a direct modeling strategy for the RMST, using leave‐one‐out jackknife pseudo‐values within a random forest regression framework. In this way, it can be employed to obtain precise estimates of both patient‐specific RMST values and confounder‐adjusted treatment contrasts. Since our method (termed “pseudo‐value random forest”, PVRF) is model‐free, RMST estimates are not affected by restrictive assumptions like the proportional hazards assumption. Particularly, PVRF offers a high flexibility in detecting relevant covariate effects from higher‐dimensional data, thereby expanding the range of existing pseudo‐value modeling techniques for RMST estimation. We investigate the properties of our method using simulations and illustrate its use by an application to data from the SUCCESS‐A breast cancer trial. Our numerical experiments demonstrate that PVRF yields accurate estimates of both patient‐specific RMST values and RMST‐based treatment contrasts.

**Abbreviations**
AFT: accelerated failure time
BMI: body mass index
CART: classification and regression trees
CI: confidence interval
DFS: disease‐free survival
ER: estrogen receptor
GEE: generalized estimation equation
HER2: human epidermal growth factor receptor 2
HR: hazard ratio
IPC: inverse‐probability‐of‐censoring
MSE: mean squared error
PFI: permutation feature importance
PH: proportional hazards
PR: progesterone receptor
PVRF: pseudo‐value random forest
RCT: randomized controlled trial
RMSE: root mean squared error
RMST: restricted mean survival time
SD: standard deviation
WRSS: weighted residual sum of squares

## 1 Introduction

During the past years, an increasing number of statisticians and applied researchers have advocated the use of the restricted mean survival time (RMST) to summarize event times in longitudinal studies [1, 2]. Defined as the area under the survival function within a time interval [0,τ], the RMST represents the expected event time between zero and the “time horizon” τ>0. In medical research, using the RMST as a summary measure offers the following specific advantages: (i) Its interpretation as the life expectancy between 0 and τ is straightforward and easily understood by both clinicians and patients [3], (ii) instead of a single time point (evaluated, e.g., by t‐year survival probabilities in cancer research), the entire survival history up to τ is reflected by the RMST, (iii) in contrast to the hazard ratio (HR) derived from Cox regression, the RMST can be used for meaningful treatment comparisons even when the proportional hazards (PH) assumption is violated [1, 4], and (iv) the RMST can be used to define estimands for the causal interpretation of treatment and interventional effects [5]. As a result, the reporting of the RMST in medical studies has become increasingly prevalent [6, 7, 8].

In addition to the calculation of absolute RMST values, *differences* between group‐wise RMST values have been suggested as a measure of treatment contrasts in longitudinal studies [6]. In medical research, treatment contrasts are often expressed and evaluated by the HR derived from a Cox PH model [1]. However, the interpretation of this type of HR is only valid if the PH assumption holds, assuming the HR to be constant over time. Thus, Stensrud and Hernán [8] recommended to supplement the reporting of HRs by summary measures directly derived from the survival function S(t)=P(T>t) (with T denoting the survival time). The RMST belongs to this class of measures, as it can be expressed as $\mu (\tau )\;=E\left[\min(T,\tau )\right]=\int_{0}^{\tau }S(t)\;dt$ and therefore directly summarizes the survival function in [0,τ]. Similarly, the RMST difference for two treatment groups A and B with survival functions $S_{A}(t)$ and $S_{B}(t)$, defined by $\mu_{A}(\tau )-\mu_{B}(\tau )\;=\;\int_{0}^{\tau }\left(S_{A}(t)-S_{B}(t)\right)dt$, can simply be interpreted as the difference in life expectancy or as a gain (or loss) in event‐free survival time before τ [3].

This paper is concerned with the estimation of RMSTs conditional on covariates $\mu (\tau |X_{i})=\int_{0}^{\tau }S(t|X_{i})\;dt,\;i=1,\ldots ,n$, from a set of n independent individuals with possibly right‐censored event times (in the following referred to as *individual RMSTs*). The covariate values are denoted by $X_{i}={\left(X_{i}^{\left(1\right)},\ldots ,X_{i}^{\left(p\right)}\right)}^{⊤}\in {\mathbb{R}}^{p}$. For ease of notation and without loss of generality, we assume all treatment and interventional variables to be included in $X_{i}$. Our method is characterized by a non‐parametric approach combining pseudo‐value modeling [9] with random forest regression [10, 11]. Using the estimated individual RMSTs, we pursue two goals: (a) To incorporate the effects of a (possibly large and interacting) set of covariates in the estimation of the RMST, and (b) to quantify and assess accuracy of treatment effect estimation through RMST differences in observational longitudinal trials.

Standard approaches to estimate individual RMSTs $\mu (\tau |X_{i})$ are the direct integration of group‐wise Kaplan–Meier curves (leading to identical RMST estimates for individuals belonging to the same treatment group) and the integration of survival functions estimated through a parametric or semi‐parametric time‐to‐event model with covariates $X_{i}$ (e.g., a Cox PH model or an accelerated failure time (AFT) model [4, 12]). Using these standard approaches, the estimation of treatment effects through RMST differences is straightforward. Previous research on RMST differences also includes the work by Royston & Parmar [1], Tian et al. [13] and Huang & Kuan [14], who developed hypothesis tests for RMST differences derived by group‐wise integration of Kaplan–Meier curves. Clearly, the covariate‐free Kaplan–Meier approach is not recommended for use in non‐randomized studies, as it ignores the effects of potential confounders on RMST differences. While integrating estimated survival functions derived from Cox PH or AFT models mitigates this problem, the validity of the resulting RMST estimates strongly depends on the correctness of the underlying model and/or distributional assumptions [15].

Instead of estimating individual RMSTs by integrating survival functions derived from time‐to‐event models, several authors have suggested to *directly* model the RMST [16, 17, 18]. In general, the idea of direct modeling approaches is to estimate unconditional individual RMSTs (without using any covariate information) and to subsequently fit a statistical model regressing these values to the covariates. Key advantages of directly modeling the RMST are less restrictive distributional assumptions as well as the straightforward interpretation of the model coefficients [1, 3, 6, 19].

In this paper, we pursue a direct approach for modeling RMST values and their differences. More specifically, the idea of our method is to derive unconditional RMST values from jackknife pseudo‐values and to regress these values to the covariates using random forests. Classical pseudo‐value regression for the RMST difference [20] is based on parametric models of the form

(1) $$\begin{array}{ll} & g[\mu (\tau |X_{i})]=\alpha +\gamma^{T}X_{i}=:\eta_{i}, \end{array}$$

with a monotonic link function g, an intercept α and covariate effects γ. Note that we suppress the dependency of α, γ and $\eta_{i}$ on τ for ease of notation. Andersen et al. [9] and Andersen & Pohar Perme [20] suggested to estimate unconditional RMST values by leave‐one‐out jackknife pseudo‐values ${\hat{\theta }}_{i}(\tau )$ defined as

(2) $$\begin{array}{ll} {\hat{\theta }}_{i}(\tau ) & =n\cdot \int_{0}^{\tau }{Ŝ}_{\text{KM}}(t)\;dt\;-\;(n-1)\cdot \int_{0}^{\tau }{Ŝ}_{\text{KM}}^{-i}(t)\;dt,\;\;\;\;i=1,\ldots ,n, \end{array}$$

where ${Ŝ}_{\text{KM}}(t)$ denotes the Kaplan–Meier estimate evaluated at t calculated on the complete data set and ${Ŝ}_{\text{KM}}^{-i}(t)$ denotes the respective Kaplan–Meier estimate calculated on the data set without individual i.

The coefficients in (1) can be estimated by a generalized estimation equation (GEE) approach, with g being the identity or the log link [9]. However, while the GEE approach yields consistent estimates (n→∞) under the assumption of random censoring [21, 22], its flexibility is limited by the restrictive specification of the main‐effects predictor $\eta_{i}$ in (2). Although more flexible effect terms (representing, e.g., interaction terms or non‐linear main effects) could be included in (1), this approach is not commonly used in practice. Often, this is due to the fact that pre‐specifying an extended version of $\eta_{i}$ requires detailed knowledge on the, usually unknown, dependency structure between the pseudo‐value outcome and the covariates. Further, the GEE approach does, in its basic form, neither incorporate any mechanism for data‐driven variable selection nor perform any other sort of regularization to reduce redundant or irrelevant information.

To address these issues, and to achieve the goals stated in (a) and (b), we propose to replace the GEE approach by a random forest regression [10]. This regression model, which uses the pseudo‐values ${\hat{\theta }}_{i}(\tau )$ as continuous outcome and which will be termed “pseudo‐value random forest” (PVRF) in the following, allows for a data‐driven selection of covariates and their interaction effects. In this way, the need to pre‐specify $\eta_{i}$ is eliminated, making PVRF a convenient method for applications involving a large number of covariates compared to the number of individuals (for instance, in medium‐sized observational studies containing many potential confounders). By applying a g‐computation formula [23, 24] to the estimated RMST values, the PVRF method further allows for the direct estimation and causal interpretation of RMST differences. To additionally facilitate interpretability of the covariate effects, we propose to use methods for interpretable machine learning, as described in Molnar [25].

The remainder of this paper is organized as follows: In Section 2, we define relevant terms and provide a detailed description of the PVRF method. Sections 3 and 4 contain the results of a simulation study investigating the properties of the PVRF method and comparing the proposed approach to established methods for RMST estimation. Section 5 presents an application of the PVRF method to data from the SUCCESS‐A study, a randomized phase III trial investigating the effects of two treatment regimens on the disease‐free survival of patients with early breast cancer [26]. Section 6 concludes with the main findings and a brief overview of related approaches.

## 2 Methods

We consider a set of n independent individuals subject to right‐censoring with covariate values $X_{i}=(X_{i}^{\left(1\right)},\ldots ,X_{i}^{\left(p\right)})$, i=1,…,n, measured at baseline. The individual survival time and censoring time are denoted by $T_{i}$ and $C_{i}$, respectively. The observed survival time is denoted by ${\tilde{T}}_{i}=\min(T_{i},C_{i})$, and the status variable $\delta_{i}=1_{\left\{C_{i}>T_{i}\right\}}$ indicates whether the i‐th individual is censored ($\delta_{i}=0$) or whether the event of interest has been observed ($\delta_{i}=1$). Following Graw et al. [21], we assume that the censoring times are independent of both the covariates and the event times.

### 2.1 Estimation and Modeling of the RMST via Pseudo‐Values

**FIGURE 1:**
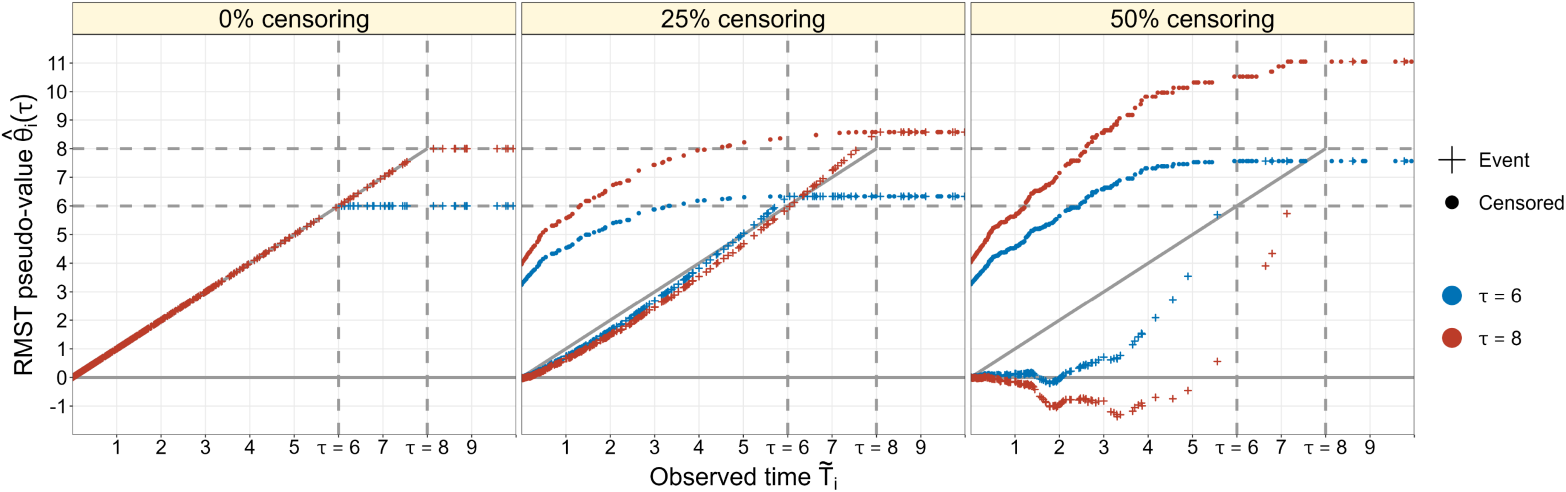
Illustration of pseudo‐values, as derived from a synthetic data set with n=500. The dashed vertical lines indicate the time horizons τ∈{6,8}. Pseudo‐values of censored and uncensored individuals are represented by dots and plus symbols, respectively (blue: τ=6, red: τ=8). In the data with no censoring (left panel), it holds that ${\hat{\theta }}_{i}(\tau )={\tilde{T}}_{i}=T_{i}$ if ${\tilde{T}}_{i}<\tau$ and ${\hat{\theta }}_{i}(\tau )=\tau$ if ${\tilde{T}}_{i}\geq \tau$. For censored individuals (dots in the middle and right panels), it is observed that ${\hat{\theta }}_{i}(\tau )>{\tilde{T}}_{i}$ for ${\tilde{T}}_{i}<\tau$, irrespective of the choice of τ and the censoring proportion. For individuals with an observed event in data sets with censoring, there is no consistent pattern regarding the dependency of pseudo‐values on ${\tilde{T}}_{i}$: At a lower censoring proportion, pseudo‐values of individuals with an observed event closely resemble the observed times (plus symbols in middle panel). In contrast, at a higher censoring proportion, pseudo‐values of individuals with an observed event are mostly lower than the observed event time and can even become negative (plus symbols in the right panel). For ${\tilde{T}}_{i}\geq \tau$, it holds that ${\hat{\theta }}_{i}(\tau )\geq \tau$ for all individuals. Consequently, there is no difference between individuals who were censored after time τ and those who were observed to experience an event after τ. Figure adapted from Andersen & Pohar Perme [20].

When using the RMST, defined as $\mu (\tau )=E\left[\min(T,\tau )\right]=\int_{0}^{\tau }S(t)\;dt$, as dependent variable in a regression model, the outcome values are given by $\mu_{i}(\tau )$ $=\min(T_{i},\tau ),i=1,\ldots ,n$. By definition, these values depend on the survival times $T_{i}$, and, due to censoring, cannot be observed for all individuals. Pseudo‐value regression overcomes this problem by replacing the partly incompletely observed outcome values with continuous (real‐valued) pseudo‐values ${\hat{\theta }}_{i}(\tau )$ that can be computed for both censored and uncensored individuals. For the RMST, the i‐th pseudo‐value at a time horizon τ is given by the right‐hand side of Equation (2). The pseudo‐values can subsequently be used as a (completely observed) imputation for the outcome variable $\mu_{i}(\tau )$ in the RMST regression model, facilitating the application of conventional modeling techniques like linear regression or trees [27]. It can be shown that the replacement of $\mu_{i}(\tau )$ with ${\hat{\theta }}_{i}(\tau )$ enables the consistent estimation of covariate effects on the RMST (see Overgaard et al. [22] for details and regularity assumptions). As seen from Figure 1, the characteristics of pseudo‐values for the RMST depend on the observed time ${\tilde{T}}_{i}$, the censoring proportion in the data set and the time horizon τ. In general, it appears hard to approximate the empirical distribution of the pseudo‐values by a parametric distribution.

As outlined in Section 1, the standard approach to pseudo‐value regression for the RMST is to use the unconditional pseudo‐values ${\hat{\theta }}_{i}(\tau )$ as outcome variable in a GEE model (see Equation (1)). The estimated coefficients of this model can be interpreted as direct effects on the RMST if g is the identity link, or on the logarithm of the RMST if g is the log link. For details on GEE estimation, we refer to Graw et al. [21].

Despite the popularity of the GEE approach, it is easily seen from (1) that the estimated RMST values are constrained to a rather restrictive linear combination of the covariate effects. Particularly, (1) does not include any interaction terms. While these terms could be pre‐specified in $\eta_{i}$, it is well known that the number of interactions grows exponentially with the number of covariates. This implies numerous coefficients to be estimated and a high variance of the GEE estimator even in cases with a moderate number of covariates. If there is no expert knowledge available to pre‐select a suitable (small) number of interaction terms, data‐driven variable selection techniques (such as forward or backward selection) could be applied. However, these algorithms usually show a high variability and are not recommended for the selection of interaction terms.

### 2.2 Random Forest Regression

To address the issues described in Section 2.1, we propose to model individual RMSTs by using the pseudo‐values ${\hat{\theta }}_{i}(\tau )$ as continuous outcome variable in a random forest regression model [10, 11]. Random forest regression is characterized by averaging estimates of multiple regression trees trained on different random subsets of the data (“ensemble” of regression trees). In this way, overfitting is avoided, and both interactions and non‐linear covariate effects are captured by the model [10].

The general idea of building a regression tree is to derive local estimates of the outcome variable by partitioning the covariate space into a set of mutually exclusive subspaces [28, 29, 30]. Beginning with the *root node* containing all individuals i=1,…,n, the idea is to successively evaluate a split criterion and to split individuals into two mutually exclusive sets termed *daughter nodes*. Each daughter node $R_{m}\subseteq \{1,\ldots ,n\}$ is further split into two daughter nodes $R_{m_{1}}\subset R_{m}$ and $R_{m_{2}}\subset R_{m}$ with $R_{m_{1}}\cap R_{m_{2}}=\emptyset$, and splitting is continued until some stopping criterion applies (see Appendix B). In each node $R_{m}$, splitting is done by selecting a split variable $X^{\left(j^{*}\right)}$, $j^{*}\in \{1,\ldots ,p\}$, and a corresponding split rule ${ℛ}_{mj^{*}}$ that optimize a pre‐defined split criterion (e.g., the mean squared error, see Hothorn et al. [29] and Greenwell [30] for details on split rules). The split criterion is evaluated on the data of the individuals in the respective node $R_{m}$. Nodes that are not further split into two daughter nodes because the stopping criterion applies are referred to as *leaf nodes*. For calculating the estimated RMST value of a single individual, the associated leaf node is determined by using the individual's covariate values and by successively applying the split rules from the root node to the leaf node. Afterwards, the RMST value is estimated by averaging the observed pseudo‐values ${\hat{\theta }}_{i}(\tau )$ in the leaf node.

Random forest regression is characterized by growing large ensembles of regression trees. In this paper, we will use 500 trees unless stated otherwise. Furthermore, we follow the recommendation by de Bin et al. [31] and grow our tree ensemble on subsamples of the complete data without replacement. Thus, each tree in the forest is grown on a different subset of the data, leading to different split rules and different RMST estimates in the leaf nodes. Additionally, only a random subset of the covariates is considered for splitting the nodes of the regression trees. We determine the size of this subset (termed “mtry”) using five‐fold cross‐validation, see Appendix B. The final RMST estimate for individual i is obtained by dropping the covariate values $X_{i}$ down to the leaf nodes of the 500 trees and by averaging the 500 tree estimates.

In the literature, there exist multiple tree building algorithms that vary in the procedure to select the split variables and the corresponding split rules. In this work, we consider two different tree‐building algorithms that will be described briefly in Sections 2.2.1 and 2.2.2: (i) Classification and regression trees (CART) [28] and (ii) conditional inference trees [29]. Correspondingly, the resulting forests will be referred to as *CART random forest* and *conditional random forest*.

#### 2.2.1 CART Random Forest

In each node $R_{m}$, the CART algorithm selects the split variable $X^{\left(j^{*}\right)}$ and the corresponding split rule ${ℛ}_{mj^{*}}$ by minimizing

(3) $$\begin{array}{l} {\text{MSE}}_{R_{mj}}=\sum_{i\in R_{m_{1}}}{\left({\hat{\theta }}_{i}(\tau )-{\bar{c}}_{1}\right)}^{2}+\sum_{i\in R_{m_{2}}}{\left({\hat{\theta }}_{i}(\tau )-{\bar{c}}_{2}\right)}^{2} \end{array}$$

over ${ℛ}_{mj}$, where ${\bar{c}}_{1}$ and ${\bar{c}}_{2}$ are the averaged pseudo‐values in the daughter nodes $R_{m_{1}}$ and $R_{m_{2}}$, respectively. Consequently, the split variable and the split rule minimizing the mean squared errors of the pseudo‐values ${\hat{\theta }}_{i}(\tau )$ in the daughter nodes are selected jointly in one optimization step. In practice, this leads the CART algorithm to favor split variables with many possible splits, implying that the algorithm is biased towards the selection of covariates with many possible splits (e.g., continuous covariates) [29].

#### 2.2.2 Conditional Random Forest

Unlike the CART algorithm, conditional inference trees [29] follow a two‐step process in each node, selecting the optimal split variable by a set of statistical hypothesis tests *before* determining the corresponding split rule. In this way, a selection bias towards covariates with many possible splits is avoided. More specifically, in the first step, the null hypotheses of independence between the covariates and the outcome values ${\hat{\theta }}_{i}(\tau )$ are tested by evaluating a set of generalized correlation coefficients $\rho_{j}$, j=1,…,p (measuring the pairwise associations between the outcome and the covariates), and by computing a permutation‐based p‐value for each covariate using the conditional distributions of transformed versions of $\rho_{j}$ under the null. Finally, the covariate with minimum p‐value in the permutation tests is selected as a split variable. Since the p‐values do not depend on the scales of the covariates, the selection procedure does not show any systematic preference towards covariates with many possible splits. For details on the definition of $\rho_{j}$ and the test procedure, we refer to Hothorn et al. [29].

The second step is to derive the split rule associated with the selected split variable $X^{\left(j^{*}\right)}$. Analog to the CART algorithm, each possible split rule leads to two possible daughter nodes $R_{m_{1}}$ and $R_{m_{2}}$. To determine the optimal split rule ${ℛ}_{mj^{*}}$, the idea is to maximize a criterion that is constructed in the same way as the generalized correlation coefficients $\rho_{j}$, this time measuring the association between the pseudo‐values and node membership. Details on the selection procedure are given in Hothorn et al. [29].

### 2.3 Evaluating RMST Differences

In medical research, a common aim is to compare subgroups of the population with regard to their survival behavior. Usually, these subgroups are defined by an intervention (e.g., treatment vs. control, see Section 5) or by the presence of a risk factor. Following Royston & Parmar [4], Uno et al. [6] and Dehbi et al. [7], we quantify differences in the survival behavior of population subgroups (in the following termed “treatment contrasts”) using differences in RMST values. In randomized controlled trials (RCTs), which usually allow for ignoring all covariates except the intervention due to the randomization procedure, treatment contrasts can simply be estimated by the differences of the average RMST values in the relevant groups. When additional covariates have to be taken into account, particularly in non‐randomized studies where the covariates usually take the roles of confounders, we propose to apply g‐computation to estimate treatment contrasts [23, 24]. More specifically, denoting the treatment variable of individual i by $X_{i}^{\left(\text{trt}\right)}$ and the respective confounders by $X_{i}^{\left(-\text{trt}\right)}$, we propose to calculate RMST differences as

(4) $$\begin{array}{ll} {\hat{\Delta }}_{i}(\tau ) & =\hat{\mu }(\tau |X_{i}^{\left(-\text{trt}\right)},X_{i}^{\left(\text{trt}\right)}=A)-\hat{\mu }(\tau |X_{i}^{\left(-\text{trt}\right)},X_{i}^{\left(\text{trt}\right)}=B)\;,i=1,\ldots ,n, \end{array}$$

where $\hat{\mu }$ denotes the RMST estimate obtained from the random forest model (see Hu et al. [32] for alternative ways to define and estimate survival treatment effects). Based on the individual RMST differences, the average treatment effect (= treatment contrast) is estimated by

(5) $$\hat{\Delta }(\tau )=\frac{1}{n}\sum_{i=1}^{n}{\hat{\Delta }}_{i}(\tau )=\frac{1}{n}\sum_{i=1}^{n}[\hat{\mu }(\tau |X_{i}^{\left(-\text{trt}\right)},X_{i}^{\left(\text{trt}\right)}=A)-\hat{\mu }(\tau |X_{i}^{\left(-\text{trt}\right)},X_{i}^{\left(\text{trt}\right)}=B)],$$

### 2.4 Pseudo‐Value Random Forest

Summarizing Sections 2.1 to 2.3, we define our proposed method (termed “pseudo‐value random forest”, PVRF) by the following steps:

1. Calculate pseudo‐values ${\hat{\theta }}_{i}(\tau ),\;i=1,\ldots ,n$, for the RMST (Equation (2)).
2. Grow a random forest using either the CART algorithm (Section 2.2.1) or conditional inference trees (Section 2.2.2).
3. Estimate RMST values conditional on covariates $\hat{\mu }(\tau |X_{i}),\;i=1,\ldots ,n$, from the fitted random forest.
4. Depending on the research question,
  - proceed analyzing estimated RMST values using interpretable machine learning techniques (see Section 5).
  - estimate treatment contrasts $\hat{\Delta }(\tau )$ from individual RMST differences ${\hat{\Delta }}_{i}(\tau ),\;i=1,\ldots ,n$ (Equation (5)).

## 3 Experiments

To investigate the performance of PVRF, we carried out a comprehensive simulation study in R (version 4.1.2 [33]). The data‐generating process was based on a time‐to‐event model with an additive combination of main and interaction effects. We analyzed the ability of PVRF to estimate RMSTs and RMST differences conditional on covariates between treatment groups in the absence and presence of two‐way interactions. To this end, we considered scenarios with time‐constant and time‐varying treatment effects. The simulation study was based on 100 Monte Carlo replications. In each replication, we generated a data set of size n=1000.

Survival times $T_{i}$, i=1,…,n, were generated from a Weibull model with scale parameter λ>0, shape parameter ν>0 and hazard function $h(t|X_{i})=\lambda \cdot \exp\left(\eta_{i}(t)\right)\cdot \nu \cdot t^{\nu -1}$, where $\eta_{i}(t)$ is the (possibly time‐dependent) linear predictor of individual i (depending on $X_{i}$, see Equation (6)). The cumulative hazard function was given by $H(t|X_{i})=\lambda \cdot \exp\left(\eta_{i}(t)\right)\cdot t^{\nu }$. The censoring times were generated independently of the survival times, using the same Weibull model with $\eta_{i}(t)=0$. The parameters λ and ν were adjusted such that the data‐generating process yielded the desired censoring proportions.

Overall, we examined four scenarios, each differing in the calculation of $\eta_{i}(t)$. Each scenario was characterized by five continuous covariates (denoted by $X_{i}^{\left(j\right)}$, j=1,…,5) and five dichotomous covariates (denoted by $X_{i}^{\left(j\right)}$, j=6,…,10). The continuous covariates followed a multivariate normal distribution with zero mean and a covariance matrix as given in Table A1 in Section A. Dichotomous covariates were independent and followed Bernoulli distributions with probability 0.5 each. In addition, we considered a dichotomous treatment variable $X_{i}^{\left(\text{trt}\right)}$ (treatment A vs. B, Bernoulli distributed with probability 0.5). The scenarios further differed in the structure of the interactions between the covariates and the strength of the treatment effects. We considered predictors of the form

(6) ηi(t)=∑j=110δjXi(j)+∑l∈{1,…,5}m∈{1,…,5}ψlmXi(l)Xi(m)+∑r∈{1,…,5}s∈{6,…,10}φrsXi(r)Xi(s)+ϑtrt(t)1{Xi(trt)=B},

where $\delta_{j}$, j=1,…,10, denote main effects of the continuous and the dichotomous covariates, $\psi_{lm}$, l,m∈{1,…,5}, represent the interaction effects between the continuous covariates, $\varphi_{rs}$, r∈{1,…,5},s∈{6,…,10}, represent the interaction effects between the continuous and the dichotomous covariates, and $\vartheta_{\text{trt}}(t)$ denotes the (possibly time‐varying) treatment effect. All main and interaction effects were sampled from a continuous uniform distribution on [−1,1]; they were generated independently of each other and were the same in all Monte Carlo replications. Furthermore, we added five independent standard normally distributed noise variables to the covariate set. These were independent of the other covariates and did not affect the predictor $\eta_{i}(t)$. In Scenarios 1 and 2, the treatment effect was time‐constant, whereas in Scenarios 3 and 4, the treatment effect changed at the transition time $t_{0}$, resulting in crossing survival curves (Figure 3). Scenarios 1 and 3 included only main effects, whereas Scenarios 2 and 4 additionally included interaction effects. Table 1 provides an overview of the four scenarios, and Figure 3 presents the group‐wise Kaplan–Meier curves for each scenario.

**TABLE 1:** Overview of the four scenarios used in the simulation study, each characterized by five continuous covariates, five dichotomous covariates, eight interaction effects (Scenarios 2 and 4), and a time‐constant (Scenarios 1 and 2) or time‐varying (Scenarios 3 and 4) treatment effect. Interaction effects not contained in the fourth column were set to zero.

Scenario	Effects of continuous covariates	Effects of dichotomous covariates	Interaction effects	Treatment effect
1	$\delta_{j}∼U(-1,1)$	$\delta_{j}∼U(-1,1)$	$\psi_{lm}=0\;\forall l,m$	$\vartheta_{\text{trt}}(t)=-2$
	j=1,…,5	j=6,…,10	$\varphi_{rs}=0\forall r,s$	
2	$\delta_{j}∼U(-1,1)$	$\delta_{j}∼U(-1,1)$	$\psi_{13},\psi_{14},\psi_{23},\psi_{25},\psi_{45}∼U(-1,1)$	$\vartheta_{\text{trt}}(t)=-2$
	j=1,…,5	j=6,…,10	$\varphi_{17},\varphi_{28},\varphi_{39}∼U(-1,1)$	
3	$\delta_{j}∼U(-1,1)$	$\delta_{j}∼U(-1,1)$	$\psi_{lm}=0\;\forall l,m$	$\vartheta_{\text{trt}}(t)=\left\{\begin{array}{ll} -2\; & t\leq t_{0}\\ 2\; & t>t_{0} \end{array}\right.$
	j=1,…,5	j=6,…,10	$\varphi_{rs}=0\forall r,s$	
4	$\delta_{j}∼U(-1,1)$	$\delta_{j}∼U(-1,1)$	$\psi_{13},\psi_{14},\psi_{23},\psi_{25},\psi_{45}∼U(-1,1)$	$\vartheta_{\text{trt}}(t)=\left\{\begin{array}{ll} -2\; & t\leq t_{0}\\ 2\; & t>t_{0} \end{array}\right.$
	j=1,…,5	j=6,…,10	$\varphi_{17},\varphi_{28},\varphi_{39}∼U(-1,1)$	

In each of the four scenarios, we considered three different censoring proportions (25%, 50%, and 75%) and five different values of the time horizon τ. The latter were determined by the 50%, 60%, 70%, 80%, and 90% quantiles of the observed times ${\tilde{T}}_{i},\;i=1,\ldots ,n$, denoted by $q_{50\%}$, $q_{60\%}$, $q_{70\%}$, $q_{80\%}$, and $q_{90\%}$, respectively. The values of τ, which were held fixed across the simulation runs, are given in Table A2 in Section A. The transition time $t_{0}$ in Scenarios 3 and 4 was set to $q_{70\%}$. In total, each scenario examined 15 combinations of censoring proportions and time horizons τ. For the values of the coefficients $\delta_{j}$, $\psi_{lm}$ and $\varphi_{rs}$, we refer to the attached R code (see Appendix B).

To evaluate performance of RMST estimates, we considered the mean squared error defined by

(7) $$\begin{array}{l} \text{MSE}=\frac{1}{n}\sum_{i=1}^{n}{\left(\hat{\mu }(\tau |X_{i})-\mu (\tau |X_{i})\right)}^{2}, \end{array}$$

where $\hat{\mu }(\tau |X_{i})$ and $\mu (\tau |X_{i})$ denote the estimated and the theoretical RMSTs, respectively, of individual i at time horizon τ. The root mean squared error (RMSE) is defined as the square root of (7). The theoretical RMST in (7) is derived as

(8) $$\begin{array}{ll} \mu (\tau |X_{i}) & =\int_{0}^{\tau }S(t|X_{i})\;dt=\int_{0}^{\tau }\exp(-H(t|X_{i}))\;dt\\ & =\left\{\begin{array}{ll} \int_{0}^{\tau }\exp(-H_{1}(t|X_{i}))\;dt,\; & \tau \leq t_{0},\\ \int_{0}^{t_{0}}\exp(-H_{1}(t|X_{i}))\;dt+\int_{t_{0}}^{\tau }\exp(-H_{1}(t_{0}|X_{i}) & \\ \;-H_{2}(t|X_{i})+H_{2}(t_{0}|X_{i}))\;dt,\; & \tau >t_{0}, \end{array}\right. \end{array}$$

where $H_{1}(t|X_{i})$ and $H_{2}(t|X_{i})$ are the cumulative hazard functions before and after the transition point $t_{0}$, respectively. Note that in Scenarios 1 and 2, the hazards are constant over time and thus $H_{1}(t|X_{i})=H_{2}(t|X_{i})$, resulting in $\mu (\tau |X_{i})=\int_{0}^{\tau }\exp(-H_{1}(t|X_{i}))dt$ for both $\tau \leq t_{0}$ and $\tau >t_{0}$. Analogously, we evaluated the accuracy of treatment effect estimates by calculating the mean squared error of the treatment effect, defined as

(9) $$\begin{array}{l} {\text{MSE}}_{\Delta }=\frac{1}{n}\sum_{i=1}^{n}{\left({\hat{\Delta }}_{i}(\tau )-\Delta_{i}(\tau )\right)}^{2}, \end{array}$$

where ${\hat{\Delta }}_{i}(\tau )$ and $\Delta_{i}(\tau )$ denote the estimated and the theoretical individual treatment effects, respectively (see Section 2.3).

In addition to evaluating the estimation accuracy of the CART and conditional random forest approaches, we compared our method to alternative modeling approaches. These were (i) a GEE pseudo‐value model with identity link (*GEE*), (ii) a GEE pseudo‐value model with log link (*GEE (log)*), (iii) a Cox PH model (*Cox*), (iv) a parametric AFT model (based on log‐transformed survival times and assuming normally distributed errors [34], *Lognormal*), and (v) a correctly specified Cox PH model (*Reference*). For the modeling approaches (i)–(iv), we specified the main effects of all continuous and dichotomous covariates (including the noise variables) but did not consider any interaction terms. The *Reference* model was specified such that it corresponded to the true data‐generating process, incorporating the *informative* (= non‐zero) main and interaction effects only (see Table 1). The *Reference* model also accounted for the time‐dependent treatment effect in Scenarios 3 and 4. This was accomplished by specifying a time‐varying stratification variable that enabled the Cox model to estimate a time‐dependent treatment effect. Consequently, *Reference* served as a lower benchmark in the RMSE and ${\text{RMSE}}_{\Delta }$ comparisons. For the *Cox*, *Lognormal* and *Reference* models, which do not directly model the RMST, estimates of the RMST were derived through the integration of the estimated survival function. Further details on the implementation of the methods are given in Appendix B.

For the main‐effects‐only Scenario 1, we expect the *Cox* and *Lognormal* models (both assuming a main effects structure) to show a better performance than the PVRF method. In contrast, we anticipate that the CART random forest and the conditional random forest approaches will outperform the *Cox*, *Lognormal*, *GEE*, and *GEE (log)* models in the scenarios with non‐zero interaction terms (Scenarios 2 and 4). Additionally, due to the time‐varying treatment effect, we expect the pseudo‐value methods to outperform the *Cox* model in Scenarios 3 and 4. Generally, we expect both the RMSE and ${\text{RMSE}}_{\Delta }$ values to increase with τ, since the RMST also rises with τ.

To evaluate the performance of the PVRF method and its comparators in a misspecified scenario, we further conducted a modified version of the previously described simulation study. In this additional study, data were simulated in the same way as before (Table 1), but one informative continuous covariate ($X^{\left(2\right)}$) and one informative dichotomous covariate ($X^{\left(7\right)}$) were excluded from the set of candidate covariates used for model fitting. As a result, all methods were provided with a reduced set of covariates. In this study, we expect both the RMSE and ${\text{RMSE}}_{\Delta }$ values to increase relative to the study using the full candidate covariate set. Additionally, we anticipate that the *Cox* and *Lognormal* models will be less advantageous in Scenario 1 compared to the PVRF method. Similar to the simulation study above, we expect the PVRF method to be superior to all comparators in Scenarios 2, 3, and 4.

## 4 Results

**FIGURE 2:**
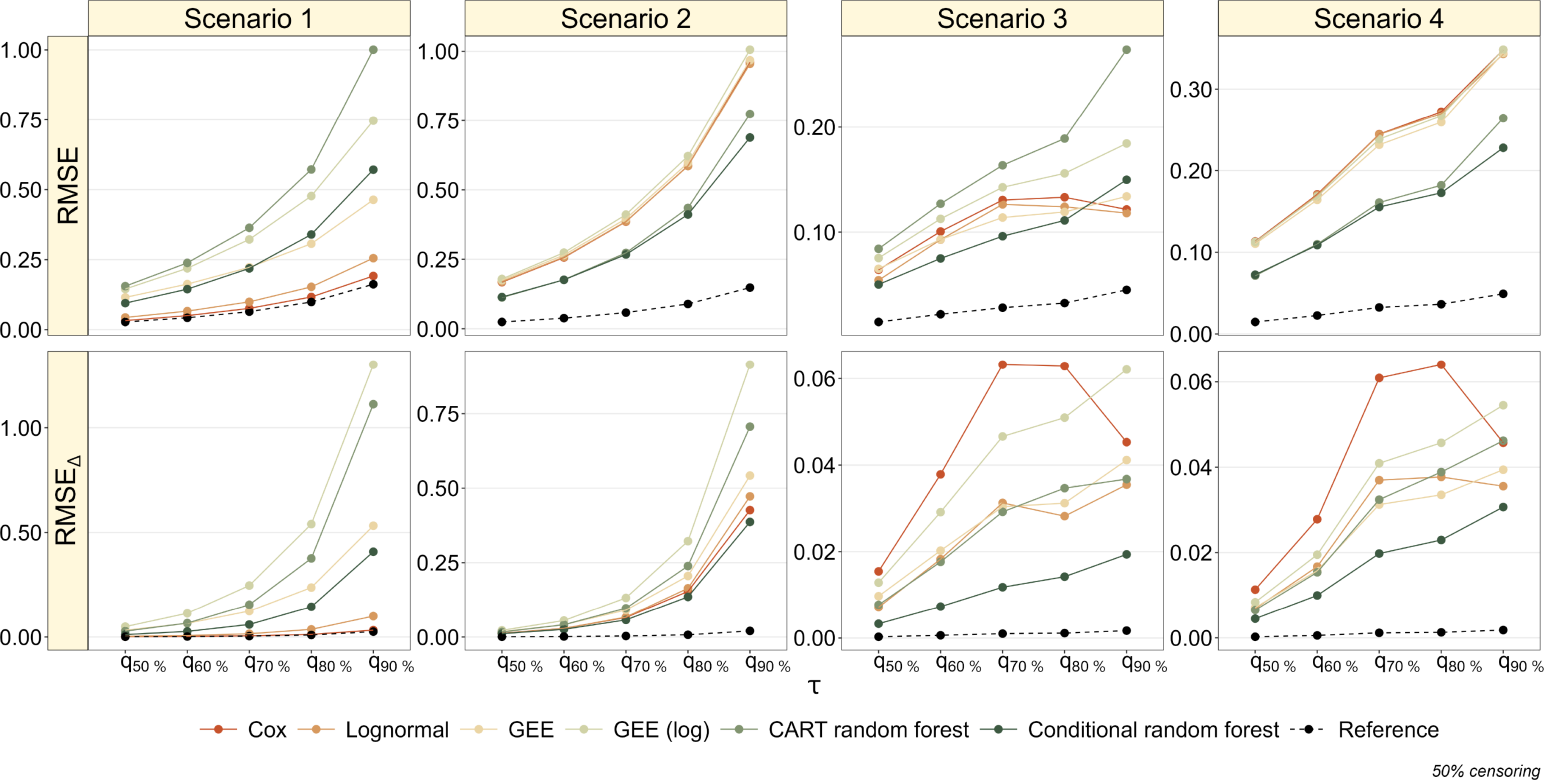
Results of the simulation study (50% censoring). The upper panels present the average RMSE (7), as obtained from the RMST estimates at different values of τ. The lower panels present the average RMSE for the treatment effect (9). The dashed black lines refer to the correctly specified Cox PH model (*Reference*).

Figure 2 summarizes the simulation results of the four scenarios at a censoring proportion of 50%. In the first scenario (main effects only, time‐constant treatment effect), both the average RMSE and the average ${\text{RMSE}}_{\Delta }$ increase with τ, as expected. This is true for all considered models. Notably, there is a clear difference in terms of RMSE between the standard modeling techniques (*Cox* and *Lognormal*) and the pseudo‐value methods (*GEE*, *GEE (log)*, CART random forest and conditional random forest), with the best performing model being the *Cox* model followed by the *Lognormal* model. This result can be explained by the fact that the *Cox* model matches the data‐generating mechanism in this scenario (except for the noise variables). Among the pseudo‐value regression methods, the conditional random forest demonstrates superior performance for $\tau \leq q_{60\%}$ followed by *GEE*, *GEE (log)* and CART random forest. However, this is no longer true when $\tau >q_{60\%}$. In terms of the RMSE for the treatment effect (${\text{RMSE}}_{\Delta }$), the *Cox* model demonstrates the best performance, in line with our expectations, followed by the *Lognormal* model. Among the pseudo‐value methods, the conditional random forest performs best with regard to treatment effect estimation, followed by the *GEE* model. The application of the log link in the GEE approach (*GEE (log)*) appears to have a negative effect on both performance measures (first column of Figure 2). Notably, the CART random forest shows inferior performance compared to the conditional random forest and to all other comparators.

In Scenario 2 (non‐zero interaction effects, time‐constant treatment effect), the average ${\text{RMSE}}_{\Delta }$ increases with τ, similar to Scenario 1. The tree‐based pseudo‐value methods, particularly the conditional random forest, perform best in terms of RMSE, having a slight advantage over the CART random forest. This result demonstrates the ability of tree‐based methods to identify and model interactions between the covariates. All other methods perform similarly in this scenario. Regarding treatment effect estimation, the conditional random forest performs best, having slight advantages over the standard *Cox* and *Lognormal* modeling techniques, as well as over the CART random forest. Although the *Cox* and *Lognormal* models do not perform well in terms of RMSE, their performance regarding treatment effect estimation is comparable to the respective performance of the tree‐based methods. On the other hand, the GEE with log link shows a poor performance in the estimation of the treatment effect, with estimates getting worse as τ increases (second column of Figure 2).

**FIGURE 3:**
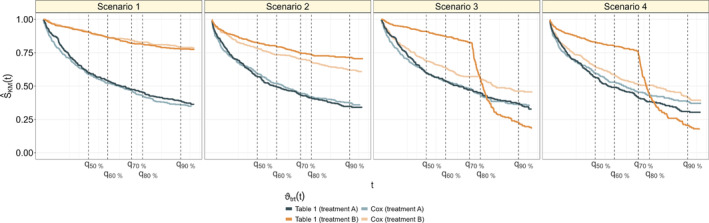
Results of the simulation study (50% censoring). The dark lines depict the Kaplan–Meier curves in the two treatment groups, as obtained from n=1000 individuals with data generated according to Table 1 (including the true treatment effects $\vartheta_{\text{trt}}(t)$). The bright lines depict the Kaplan–Meier curves derived from data generated according to Table 1 but including the time‐constant average treatment effect estimated by the *Cox* method instead of the true treatment effect (Scenario 1: ${\hat{\vartheta }}_{\text{trt}}^{\text{Cox}}(t)=-2.10$, Scenario 2: ${\hat{\vartheta }}_{\text{trt}}^{\text{Cox}}(t)=-1.26$, Scenario 3: ${\hat{\vartheta }}_{\text{trt}}^{\text{Cox}}(t)=-0.38$, Scenario 4: ${\hat{\vartheta }}_{\text{trt}}^{\text{Cox}}(t)=-0.33$).

In Scenario 3 (main effects only, time‐dependent treatment effect), the average RMSE values of the standard modeling techniques *Cox* and *Lognormal* do not monotonically increase with τ, as in Scenarios 1 and 2. Instead, there is a turning point in the RMSE values at $\tau =t_{0}=q_{70\%}$. This can be explained as follows: As seen from Figure 3, the *Cox* model assumes a time‐constant treatment effect, implying that the effect of treatment B is underestimated when $t\leq t_{0}=q_{70\%}$. This in turn leads to strongly biased RMST estimates. On the other hand, the *Cox* model overestimates the effect of treatment B when $t>t_{0}=q_{70\%}$. Consequently, as RMST estimates are derived by the area under the survival curve up to τ, the part of the area under the survival curve that is not included in the RMST estimates for $t\leq t_{0}=q_{70\%}$ is compensated by the excess area under the estimated survival curve for $t>t_{0}=q_{70\%}$. As a result, the *Cox* model yields decreasing RMSE values for $\tau >t_{0}=q_{70\%}$. For the *Lognormal* model, analog observations can be made. In contrast, the pseudo‐value methods exhibit increasing RMSE values with rising τ, as expected. The conditional random forest demonstrates superior performance compared to the *Cox* and *Lognormal* models with respect to the RMSE for $\tau \leq t_{0}$. On the other hand, as the RMSE values obtained from the *Cox* and *Lognormal* models decrease for $\tau >t_{0}$, these methods perform better than the conditional random forest at $\tau =q_{90\%}$. Notably, the CART random forest shows inferior performance compared to all other methods, which is likely due to its selection bias towards (possibly non‐informative) continuous covariates. Regarding treatment effect estimation, the ${\text{RMSE}}_{\Delta }$ values obtained from the pseudo‐value methods increase with τ. In contrast, the ${\text{RMSE}}_{\Delta }$ values obtained from the *Cox* model increase for $\tau \leq t_{0}=q_{70\%}$ but decrease for $\tau >t_{0}=q_{70\%}$. Again, this is due to the underestimation (overestimation) of the effect of treatment B for $t\leq t_{0}=q_{70\%}$ ($t>t_{0}=q_{70\%}$). The conditional random forest consistently performs best, regardless of the value of τ, confirming its ability to capture the time‐dependent treatment effect (third column of Figure 2).

**FIGURE C1:**
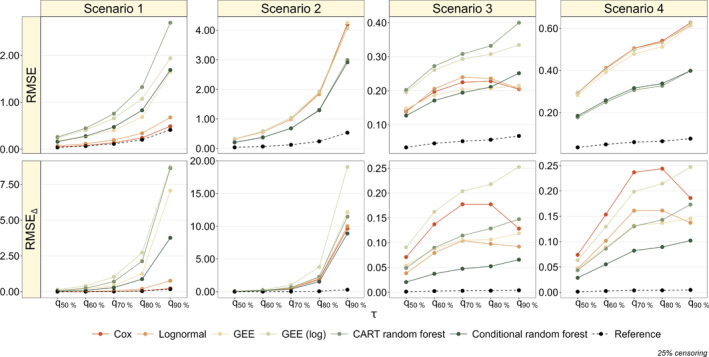
Results of the simulation study (25% censoring). The upper panels present the average RMSE (7), as obtained from the RMST estimates at different values of τ. The lower panels present the average RMSE for the treatment effect (9). The dashed black lines refer to the correctly specified Cox PH model (*Reference*).

**FIGURE C2:**
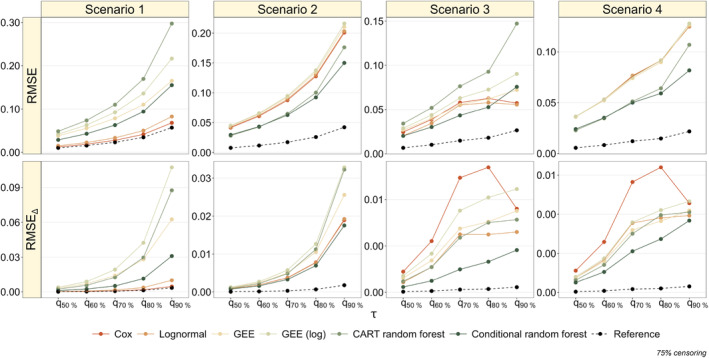
Results of the simulation study (75% censoring). The upper panels present the average RMSE (7), as obtained from the RMST estimates at different values of τ. The lower panels present the average RMSE for the treatment effect (9). The dashed black lines refer to the correctly specified Cox PH model (*Reference*).

In the presence of interaction effects, as in Scenario 4 (non‐zero interaction effects, time‐dependent treatment effect), there is a clear advantage of the tree‐based methods (CART and conditional random forest) with respect to the RMSE. Regarding treatment effect estimation, the conditional random forest performs consistently best with respect to ${\text{RMSE}}_{\Delta }$ across all time horizons τ (fourth column of Figure 2). These results highlight the ability of the conditional random forest to model interaction effects and to capture time‐dependent treatment effects simultaneously. As seen from Figure 2, the time‐dependent treatment effect influences the trend of the ${\text{RMSE}}_{\Delta }$ values of the *Cox* and *Lognormal* models, similar to Scenario 3, but not the trend of the respective RMSE values. The results obtained for 25% and 75% censoring are similar to the results shown in Figure 2. They are presented in Figures C1 and C2 in Appendix C.

**FIGURE D1:**
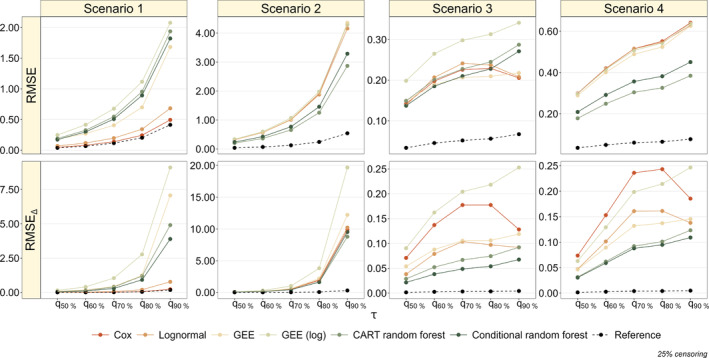
Results of the simulation study (25% censoring). The upper panels present the average RMSE (7), as obtained from the RMST estimates at different values of τ. The lower panels present the average RMSE for the treatment effect (9). All RMSE and ${\text{RMSE}}_{\Delta }$ values were obtained by applying the model fits from Section 4 to independent data sets of size $n_{\text{test}}=1000$ each that were generated according to Scenarios 1–4. The dashed black lines refer to the correctly specified Cox PH model (*Reference*).

**FIGURE D2:**
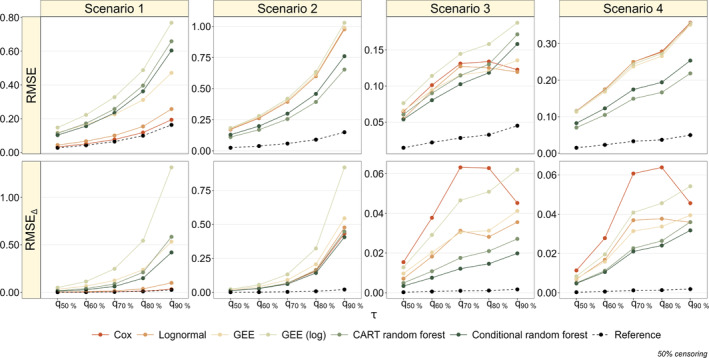
Results of the simulation study (50% censoring). The upper panels present the average RMSE (7), as obtained from the RMST estimates at different values of τ. The lower panels present the average RMSE for the treatment effect (9). All RMSE and ${\text{RMSE}}_{\Delta }$ values were obtained by applying the model fits from Section 4 to independent data sets of size $n_{\text{test}}=1000$ each that were generated according to Scenarios 1–4. The dashed black lines refer to the correctly specified Cox PH model (*Reference*).

**FIGURE D3:**
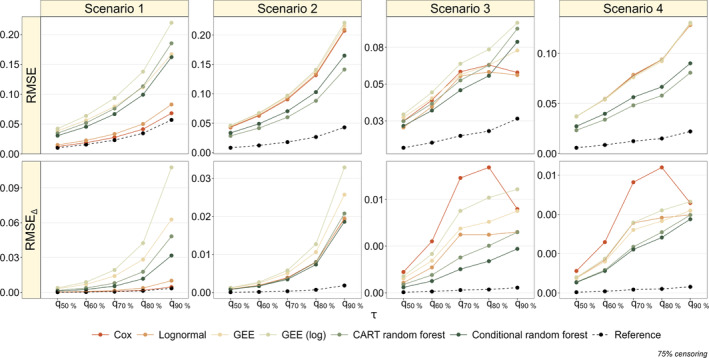
Results of the simulation study (75% censoring). The upper panels present the average RMSE (7), as obtained from the RMST estimates at different values of τ. The lower panels present the average RMSE for the treatment effect (9). All RMSE and ${\text{RMSE}}_{\Delta }$ values were obtained by applying the model fits from Section 4 to independent data sets of size $n_{\text{test}}=1000$ each that were generated according to Scenarios 1–4. The dashed black lines refer to the correctly specified Cox PH model (*Reference*).

While our primary focus was on evaluating the performance of the PVRF method in estimating RMST values conditional on covariates, we also explored the generalizability of our findings to unseen data, that is, data that were not used for model fitting. The RMSE and RMSE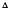 values derived on unseen data can be found in Figures D1, D2, D3 in Appendix D. In summary, we observed similar results as for the RMSE and RMSE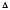 values obtained from the data used for model fitting, except for the CART random forest, which showed an improved performance.

**FIGURE 4:**
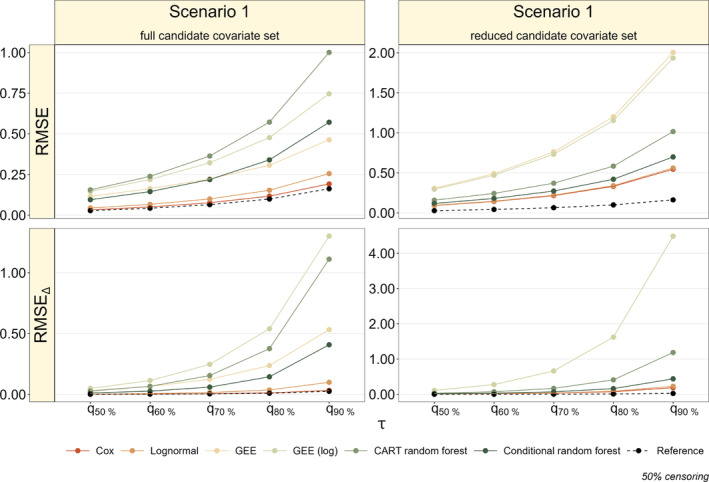
Results of the additional simulation study (50% censoring, main‐effects‐only Scenario 1, *Cox* and *Lognormal* models expected to perform well). The left panels show the average RMSE and RMSE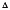 values obtained from the full candidate covariate set, whereas the right panels show the respective values obtained from the reduced candidate covariate set without $X^{\left(2\right)}$ and $X^{\left(7\right)}$. Note the different scalings of the y‐axes in the left and right panels.

**FIGURE E1:**
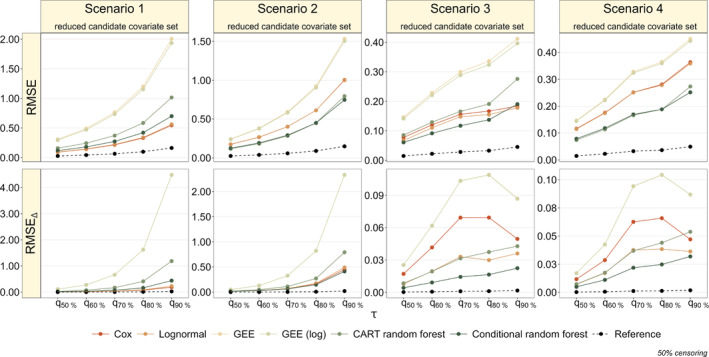
Results of the simulation study (50% censoring). The upper panels present the average RMSE (7), as obtained from the RMST estimates at different values of τ. The lower panels present the average RMSE for the treatment effect (9). All RMSE and ${\text{RMSE}}_{\Delta }$ values were obtained by applying the methods from Section 3 with the reduced candidate covariate set (excluding $X^{\left(2\right)}$ and $X^{\left(7\right)}$). The dashed black lines refer to the correctly specified Cox PH model (*Reference*).

Figure 4 presents a comparison of the results from Scenario 1 using the full and reduced candidate covariate sets at 50% censoring. As expected, the average RMSE and RMSE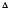 values increase when the reduced candidate covariate set, excluding $X^{\left(2\right)}$ and $X^{\left(7\right)}$, is used for the modeling approaches. Furthermore, the differences between the *Cox*, *Lognormal*, and PVRF methods decrease, suggesting that the models exhibit a more similar performance (in terms of both RMSE and RMSE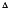) than when the full candidate covariate set is used. Put differently, the advantages of the *Cox* and *Lognormal* models are way less pronounced when $X^{\left(2\right)}$ and $X^{\left(7\right)}$ are excluded from the set of candidate covariates, indicating a higher stability of the conditional random forest. In Scenarios 2, 3 and 4, the conditional random forest still outperforms all other methods when $X^{\left(2\right)}$ and $X^{\left(7\right)}$ are removed from the candidate covariate set (see Figure E1 in Appendix E).

In summary, when considering scenarios with interactions and/or time‐varying treatment effects, we find the conditional random forest to perform better than standard modeling approaches in estimating the RMST and treatment effects. The value of τ relative to the transition time $t_{0}$ significantly impacts the performance of the standard modeling techniques *Cox* and *Lognormal*, especially in the scenarios with time‐varying treatment effects. In the main‐effects‐only scenarios, the conditional random forest still performs well when some information is omitted from the set of candidate covariates. Regarding the estimation of treatment effects, the conditional random forest performed considerably better than the CART random forest (both on the data used for model fitting and on unseen data).

## 5 Application

To illustrate the PVRF approach, we applied the conditional random forest to data from the multicenter randomized phase III SUCCESS‐A trial (NCT02181101). SUCCESS‐A enrolled 3 754 patients with a primary invasive breast cancer between September 2005 and March 2007 [26]. Study participants were randomly assigned in a 1:1 ratio to one of two treatment arms, which received either standard chemotherapy (control group) or standard chemotherapy with the addition of gemcitabine (interventional group). For details on the inclusion/exclusion criteria and the design of the study see de Gregorio et al. [26].

The primary aim of the SUCCESS‐A trial was to compare the two treatment arms with respect to disease‐free survival (DFS), defined as the period from the date of randomization to the earliest date of disease progression (distant metastases, local and contra local recurrence, and secondary primary tumors) or death from any cause [26, 35]. Here, we present the results of a secondary analysis that considered DFS as the outcome of interest. Note that the definition of DFS includes death from any cause. Accordingly, we did not consider death as a competing event.

Patients were censored at the last date at which they were known to be disease‐free, resulting in an event proportion of 12.2% (458 events in 3 754 patients). The maximum observation time was 5.5 years (6 months of chemotherapy followed by 5 years of follow‐up; median 5.2 years, first quartile 3.7 years, third quartile 5.5 years). Patient characteristics included age at randomization (*age*, in years), body mass index (*BMI*, in $kg/m^{2}$) and menopausal status (*meno*, two categories, pre‐/post‐menopausal) as well as information on the tumor, including stage (*stage*, four categories, pT1/pT2/pT3/pT4), grade (*grade*, three categories, G1/G2/G3), lymph node status (*nodal status*, two categories, pN0/pN+), type (*type*, three categories, ductal/lobular/other) and receptor status of estrogen (*ER*), progesterone (*PR*), and *HER2* (two categories each, negative/positive). A descriptive summary of the variables is given in Table F1 in Section F. Patients with missing values in any of the considered covariates were excluded from our analysis. The analyzed data comprised 3 652 patients.

The main aim of our analysis was to model the RMST for DFS at τ=5 years, corresponding to the length of the follow‐up period. To this end, we applied the conditional random forest, the *Cox* model and the *GEE* model to the SUCCESS‐A study data. The covariates were defined by the treatment (control/intervention) and the ten patient/tumor characteristics described above. The accuracy of the models was measured by the weighted residual sum of squares (WRSS), an inverse‐probability‐of‐censoring (IPC) weighted error measure [36] and by a 95% normal bootstrap confidence interval (CI, 1 000 repetitions). The WRSS is defined as

(10) $$\begin{array}{l} \text{WRSS}=\frac{1}{n}\sum_{i}{\left(\min({\tilde{T}}_{i},\tau )-\hat{\mu }(\tau |X_{i})\right)}^{2}\cdot {\hat{w}}_{i}, \end{array}$$

with $\hat{\mu }(\tau |X_{i})$ denoting the estimated RMST for individual i. The IPC weights ${ŵ}_{i}$ are defined by ${ŵ}_{i}=1\{{\tilde{T}}_{i}\leq \tau \}\cdot \delta_{i}\;/\;Ĝ({\tilde{T}}_{i^{-}}|X_{i})+1\{{\tilde{T}}_{i}>\tau \}\;/\;Ĝ(\tau |X_{i})$, where Ĝ is a consistent estimator of the censoring survival function. In this work, we use the Kaplan–Meier method to estimate Ĝ. The average treatment effect (measured in days, control vs. interventional group) was calculated as

(11) $$\begin{array}{ll} \hat{\Delta }(\tau ) & =\frac{1}{n}\sum_{i}\left[\hat{\mu }(\tau |X_{i}^{\left(-trt\right)},X_{i}^{\left(trt\right)}=\text{control})\right.\\ & \;\left.-\hat{\mu }(\tau |X_{i}^{\left(-trt\right)},X_{i}^{\left(trt\right)}=\text{interventional})\right]. \end{array}$$

To enhance the interpretability of the conditional random forest, we computed the permutation feature importance (${\text{PFI}}_{j}$) along with a 95% normal bootstrap CI and Shapley values for each covariate [25]. ${\text{PFI}}_{j}$ is defined as the ratio of the WRSS with $\hat{\mu }(\tau |X_{i})$ derived from the fitted model but using permuted values of the j‐th covariate (numerator), and the WRSS with $\hat{\mu }(\tau |X_{i})$ calculated as usual (denominator, see Equation (B1)). Thus, higher ${\text{PFI}}_{j}$ values indicate a higher importance of the j‐th covariate for estimating the RMST. Local Shapley values were derived for 1 000 randomly selected patients. A high absolute local Shapley value indicates a high importance of the respective covariate in the estimation of the RMST.

**FIGURE 5:**
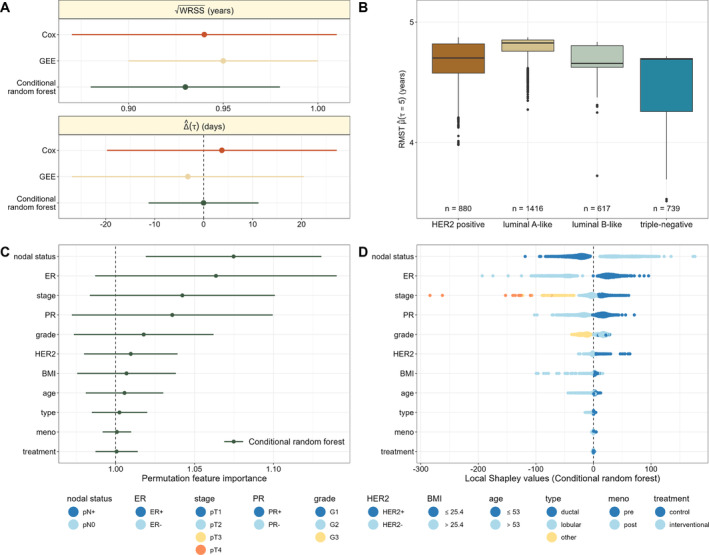
Analysis of DFS in the SUCCESS‐A study. The four panels present the results obtained from the *Cox*, *GEE* and conditional random forest methods. Panel (A) shows the square root of the WRSS (measured in years) and the treatment effect $\hat{\Delta }(\tau )$ (measured in days). The dots represent values of $\sqrt{\text{WRSS}}$ and $\hat{\Delta }(\tau )$ derived by the complete cohort, while the bars refer to 95% bootstrap confidence intervals for $\sqrt{\text{WRSS}}$ and $\hat{\Delta }(\tau )$. Panel (B) shows the estimated RMST values in patient groups defined by molecular tumor subtypes, as obtained from the conditional random forest (see Table F2). Panel (C) shows the permutation feature importance of each covariate for the conditional random forest. The dots represent ${\text{PFI}}_{j}$ values and the bars refer to the respective 95% bootstrap confidence intervals. Panel (D) presents local Shapley values for each covariate, as obtained by evaluating the conditional random forest estimates in 1 000 randomly selected patients. Each dot corresponds to one patient. The color codings used in Panel (D) are presented in the bottom row of the figure.

The results of our analysis are presented in Figure 5. They show that the conditional random forest detected several established prognostic factors and subgroups, which have been consistently reported in the literature and have also entered treatment guidelines for primary breast cancer [37, 38]. Regarding the WRSS, the conditional random forest ($\sqrt{\text{WRSS}}=0.93$ years, 95% CI: 0.88 to 0.98 years) performs best, followed by the *Cox* model ($\sqrt{\text{WRSS}}$ = 0.94 years, 95% CI: 0.87 to 1.01 years) and the *GEE* approach ($\sqrt{\text{WRSS}}$ = 0.95 years, 95% CI: 0.90 to 1.00 years, Figure 5A). The average treatment effect $\hat{\Delta }(\tau )$ estimated by the conditional random forest is close to zero (−0.01 days, 95% CI: −11.22 to 11.21 days), indicating no advantage of any of the two groups. This supports the results found by de Gregorio et al. [26], who concluded that the interventional treatment does not improve survival in patients with high‐risk early breast cancer. In contrast, the *Cox* model indicates a slight advantage of the control group ($\hat{\Delta }(\tau )=3.73$ days, 95% CI: −19.74 to 27.20 days) while the *GEE* model indicates a slight advantage of the interventional group ($\hat{\Delta }(\tau )=-3.22$ days, 95% CI: −26.92 to 20.49 days). Note, however, that the treatment difference is measured in days, so none of the obtained differences can be considered clinically relevant.

Figure 5B visualizes the RMST values at τ=5 years in patient groups defined by molecular tumor subtypes [39]. More specifically, *HER2 positive* patients are characterized by *HER2* positive tumors (regardless of *ER* status, *PR* status and *grade*). *HER2* negative tumors are further classified into *luminal A‐like* tumors (*HER2* negative, *ER* and/or *PR* status positive, *grade* G1 or G2), *luminal B‐like* tumors (*HER2* negative, *ER* and/or *PR* status positive, *grade* G3), and *triple‐negative* tumors (*HER2*, *ER* and *PR* status negative and any *grade*, see Table F2). According to Figure 5B, the high‐risk *triple‐negative* group has the lowest estimated RMST values (mean (SD): 4.46 (0.24) years), which is consistent with findings in the literature [40]. Additionally, when comparing the *luminal A‐like* and *luminal B‐like* subgroups, there appears to be a slight advantage (corresponding to higher estimated RMST values) of patients with tumor *grade* G1 (*luminal A‐like*, 4.78 (0.10) years) compared to those with tumor *grade* G2 or G3 (*luminal B‐like*, 4.68 (0.12) years). Again, this result is in line with previous findings in the literature [40]. The comparison of *luminal A‐like*, *luminal B‐like* and *triple‐negative* confirms the ability of the conditional random forest to identify interactions between hormone receptor status and grade.

As illustrated in Figure 5C, the ${\text{PFI}}_{j}$ values obtained from the conditional random forest identify *nodal status* as the most influential covariate in the estimation of the RMST. The strong influence of *nodal status* on DFS has previously been reported by Senkus et al. [38]. Other important covariates (in terms of ${\text{PFI}}_{j}$) are *ER*, *stage*, *PR*, *grade*, *HER2* and *BMI*. Notably, all other covariates appear to have negligible importance in estimating RMST values by the conditional random forest, including *treatment*. This result is in line with the findings of de Gregorio et al. [26].

**FIGURE F1:**
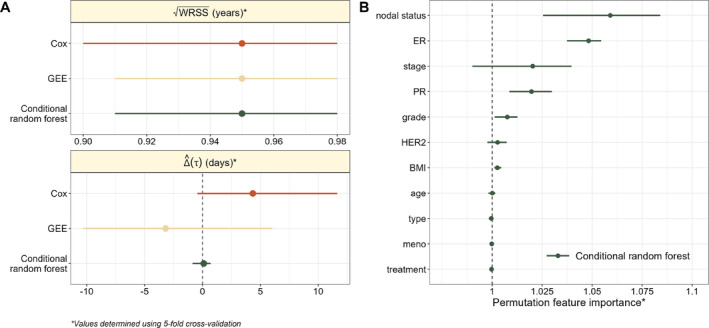
Five‐fold‐cross‐validated analysis of DFS in the SUCCESS‐A study. Panel (A) shows the square root of the WRSS (measured in years) and the treatment effect $\hat{\Delta }(\tau )$ (measured in days). The dots represent the five‐fold cross‐validated values of $\sqrt{\text{WRSS}}$ and $\hat{\Delta }(\tau )$, while the bars refer to the respective ranges of $\sqrt{\text{WRSS}}$ and $\hat{\Delta }(\tau )$ in the five folds. Panel (B) shows the permutation feature importance of each covariate for the conditional random forest. The dots represent the five‐fold cross‐validated values of PFI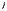 and the bars refer to the respective ranges in the five folds of the cross‐validation procedure.

The local Shapley values in Figure 5D are also in line with previous findings in the literature [38, 40, 41] and with the ${\text{PFI}}_{j}$ values in Figure 5C. As seen from Figure 5D, lymph node positive patients (*nodal status* = pN+) exhibit higher risk of recurrence or death, reflected by negative local Shapley values of these patients. Furthermore, the high Shapley values for *ER* confirm the importance of this covariate in adjuvant hormonal and chemotherapy. The survival advantage of *ER* positive patients [41] is reflected by positive Shapley values for this group. Conversely, negative Shapley values are observed for *ER* negative, *PR* negative, and *HER2* negative patients, which is consistent with lower estimated RMST values for the *triple‐negative* group in Figure 5B [40]. Likewise, the difference in estimated RMST values between *luminal A‐like* and *luminal B‐like* patients is confirmed by the respective local Shapley values: Patients with *grade* G1 and G2 have a positive contribution to the estimated RMST values, while patients with *grade* G3 contribute negatively. Furthermore, the local Shapley values accurately reflect the hierarchy of tumor stages: Tumor stage pT1 (best prognosis for DFS) has a positive contribution to the estimated RMST, whereas tumor stages pT2 to pT4 have increasingly negative contributions. Shapley values for *treatment* spread around 0 in both groups, suggesting neither a positive nor a negative contribution of the treatment to the RMST. Again, this result is consistent with the findings of de Gregorio et al. [26]. In addition to the bootstrapped estimates, we evaluated cross‐validated values of WRSS and PFI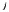. As seen from Figure F1 in Section F, these values are very similar to those obtained from the bootstrap procedure.

## 6 Discussion

During the past years, the restricted mean survival time has become an increasingly popular measure for summarizing individual event times in medical studies. Compared to other established measures like the hazard ratio, the RMST is derived from survival probabilities measured at the untransformed risk scale, thereby avoiding interpretability and collapsibility issues in the comparison of interventional groups [42, 43]. As a consequence, the RMST is considered a valid survival estimand for the causal interpretation of treatment contrasts in clinical and observational trials [5, 44].

In this work, we proposed the pseudo‐value random forest (PVRF) method, which is a non‐parametric approach for the quantification of treatment effects by group‐specific RMST values. Instead of estimating RMST values from (semi‐)parametric models like Cox or AFT regression, the PVRF method combines unconditional pseudo‐value RMST estimation with the subsequent fitting of a random forest. Except for the random censoring assumption, both components of our method (pseudo‐values and random forests) require minimal assumptions on the data‐generating process. While unconditional pseudo‐values are based on non‐parametric Kaplan–Meier estimates, random forest regression is a model‐free algorithm allowing for variable selection and requiring no prior assumptions on the structure of the covariate effects. As a result, the PVRF method is particularly suited for incorporating subgroup characteristics, non‐linearities, and higher‐order interactions affecting individual RMST values. In non‐randomized studies, this approach is particularly useful when treatment effects need to be corrected for higher‐dimensional sets of confounders, allowing for the estimation of causal contrasts via g‐computation. Furthermore, our method enables model‐free comparisons of treatment and control groups in randomized trials. Regarding the latter, we demonstrated that PVRF is able to capture time‐dependent treatment effects in a data‐driven way (see Section 3, where PVRF performed better than (semi‐)parametric approaches in the scenarios with crossing survival curves). Methods to adjust RMST estimation for covariate‐dependent censoring have been studied in Rong et al. [15].

In our numerical studies, we observed that the conditional random forest (correcting for a possible selection bias towards covariates with many possible splits) showed a better performance in terms of RMSE than the traditional CART random forest approach. This finding was particularly evident in the estimation of treatment contrasts, where conditional random forests outperformed CART in all scenarios. We therefore recommend to prefer conditional random forests over CART random forests when the aim is to estimate treatment effects from data with heterogeneous covariate types.

An important topic for future research is the development of hypothesis tests and confidence intervals for PVRF‐based RMST differences. Previous research in this field [1, 13, 14] has mainly focused on hypothesis tests for RMST differences derived by group‐wise integration of Kaplan–Meier curves (not incorporating additional covariates). Tian et al. [13] compared RMST‐based tests to HR‐based tests in the context of randomized clinical trials, demonstrating that RMST‐based tests outperformed their HR‐based counterparts in scenarios where the PH assumption is violated. It would be interesting to conduct analog studies for pseudo‐value‐based tests of RMST differences, which, to the best of our knowledge, have not yet been explored thus far. In our analysis of the SUCCESS‐A study data (Section 5), we constructed confidence intervals for treatment contrasts using bootstrap methods, along the lines of Hernán & Robins [45], Chapter 13.

A general issue in the estimation of RMST values is the choice of a suitable time horizon τ. While choosing a small value of τ may discard a large proportion of the data and will therefore result in a potential loss of information, estimation of the RMST may no longer be possible if τ becomes too large [46]. General recommendations on the choice of τ, have, for instance, been made by Tian et al. [46]: Before data collection (for instance, in the course of planning a clinical trial), it is advisable to pre‐select τ based on clinical and feasibility considerations. If pre‐selection of τ is not possible (e.g., when the analysis is conceived after data collection), Tian et al., suggest to explore a data‐dependent time window for τ and to select the time horizon based on the empirical behavior of the observed times in this window (e.g., by computing quantiles of $\tilde{T}$, as done in our simulations). Alternatively, the RMST could be modeled as a function of τ, as suggested by Zhong & Schaubel [47].

We finally note that pseudo‐value‐based RMST modeling is not restricted to the use of random forest regression. In this work, we focused on random forests because this method is considered to be “among the best “off‐the‐shelf” supervised learning methods that are available” [48]. In particular, random forests are known to perform well on medium‐sized data (as often encountered in medical applications), with several efficient software implementations being available [49]. However, it is of course possible to extend our approach to other statistical modeling or machine learning techniques, e.g., to gradient boosting [27] or deep neural networks [32, 50, 51].

## Author Contributions

A.S and M.S conceived and designed the project. A.S., V.B., and M.S. analyzed and interpreted the results. A.S. and M.S. drafted the manuscript. All authors reviewed the results and approved the final version of the manuscript.

## Disclosure

The authors have nothing to report.

## Conflicts of Interest

The authors declare no conflicts of interest.

## Supporting information


**Data S1.** Supporting Information.

## Data Availability

The data that support the findings of this study are available on request from the corresponding author. The data are not publicly available due to privacy or ethical restrictions.

## Appendix A Simulation Study

### Covariance Matrix of the Continuous Covariates

**TABLE A1:** Covariance matrix of the continuous covariates $X^{\left(j\right)}$, j=1,…,5.

	$X^{\left(1\right)}$	$X^{\left(2\right)}$	$X^{\left(3\right)}$	$X^{\left(4\right)}$	$X^{\left(5\right)}$
$X^{\left(1\right)}$	1.00	−0.08	−0.47	0.73	−0.44
$X^{\left(2\right)}$	−0.08	1.00	0.85	−0.05	−0.31
$X^{\left(3\right)}$	−0.47	0.85	1.00	−0.38	−0.33
$X^{\left(4\right)}$	0.73	−0.05	−0.38	1.00	−0.37
$X^{\left(5\right)}$	−0.44	−0.31	−0.33	−0.37	1.00

### Values of the Time Horizon τ

**TABLE A2:** Time horizons τ used in the simulation study.

Scenario	Censoring proportion	$q_{50\%}$	$q_{60\%}$	$q_{70\%}$	$q_{80\%}$	$q_{90\%}$
1	25%	1.72	2.81	4.64	8.08	16.95
	50%	1.09	1.56	2.23	3.28	5.24
	75%	0.40	0.55	0.74	1.02	1.52
2	25%	1.37	2.38	4.26	8.19	19.64
	50%	0.78	1.15	1.68	2.51	4.07
	75%	0.23	0.32	0.44	0.62	0.92
3	25%	1.41	1.83	2.05	2.23	2.74
	50%	0.64	0.88	1.07	1.22	1.65
	75%	0.31	0.42	0.55	0.64	0.86
4	25%	1.22	1.67	2.03	2.19	2.77
	50%	0.52	0.74	1.01	1.14	1.57
	75%	0.20	0.27	0.36	0.43	0.60

*Note*: Note that $q_{70\%}$ is approximately equal to the transition time $t_{0}$ in Scenarios 3 and 4.

## Appendix B Specification and Implementation of the Methods

The simulation study and the application were carried out in R, version 4.1.2 [33]. Data for the simulation study were generated using the R package simstudy, version 0.7.1 [52]. Pseudo‐values for the RMST, as defined in Equation (2), were calculated using the pseudomean function of the R package pseudo, version 1.4.3 [53].

The CART random forest was implemented using the R package ranger, version 0.15.1 [49]. The number of trees was set to 500. Data for tree building was sampled without replacement from the complete data using a sampling fraction of 0.632. The number of candidate split variables in each node (“mtry”) was tuned using five‐fold cross validation. In each tree, the minimum number of observations required to perform an additional split was set to 5. In order to avoid overoptimism in the cross‐validation procedure, we computed separate sets of pseudo‐values in each of the training and test folds. There were no restrictions on the tree depth and the minimum number of observations in the leaf nodes.

The conditional random forest was implemented using cforest function of the R package partykit, version 1.2.20 [54]. The number of trees was set to 500. By default, cforest implements sampling without replacement from the complete data, using a sampling fraction of 0.632. The number of candidate split variables in each node was tuned using five‐fold cross validation. In order to avoid overoptimism in the cross‐validation procedure, we computed separate sets of pseudo‐values in each of the training and test folds. In each tree, a minimum of 20 observations was required to perform a split, and each leaf node was required to contain a minimum of 7 observations. There was no restriction on the depth of the trees.

The *Lognormal*, *Cox*, and *Reference* methods were implemented using the R package survival, version 3.5.7 [55]. *GEE* and *GEE (log)* were implemented using the R package geepack, version 1.3.9 [56].

Permutation feature importance values were calculated as

(B1)
$$\begin{array}{l} {\text{PFI}}_{j}=\frac{{\text{WRSS}}^{\text{perm},j}}{{\text{WRSS}}^{\text{orig}}}, \end{array}$$

where ${\text{WRSS}}^{\text{orig}}$ denotes the WRSS calculated from the unpermuted data and ${\text{WRSS}}^{\text{perm},j}$ denotes the respective WRSS calculated from data with randomly permuted values of the j‐th covariate. Local Shapley values were calculated using the R package iml, version 0.11.2 [57].

The R‐code for the simulation study is available at https://www.imbie.uni‐bonn.de/cloud/index.php/s/6gmJQmayFAMJZHk.

## Appendix F Application

### Patient and Tumor Characteristics of the SUCCESS‐A Study Data

**TABLE F1:** Descriptive summary of the SUCCESS‐A study data.

Characteristic		Patients (*n* = 3 754)
Age (years)	Mean (SD)	53.5 (10.5)
	Median [Min, Max]	53.0 [21.0, 86.0]
BMI ($kg/m^{2}$)	Mean (SD)	26.3 (5.03)
	Median [Min, Max]	25.4 [15.4, 53.4]
Tumor stage	pT1	1552 (41.3%)
	pT2	1929 (51.4%)
	pT3	198 (5.3%)
	pT4	52 (1.4%)
	Missing	23 (0.6%)
Tumor grade	G1	176 (4.7%)
	G2	1783 (47.5%)
	G3	1773 (47.2%)
	Missing	22 (0.6%)
Lymph node status	pN+	2452 (65.3%)
	pN0	1273 (33.9%)
	Missing	29 (0.8%)
Tumor type	ductal	3060 (81.5%)
	lobular	419 (11.2%)
	other	253 (6.7%)
	Missing	22 (0.6%)
ER	ER‐	1252 (33.4%)
	ER+	2481 (66.1%)
	Missing	21 (0.6%)
PR	PR‐	1525 (40.6%)
	PR+	2205 (58.7%)
	Missing	24 (0.6%)
HER2	HER2‐	2787 (74.2%)
	HER2+	883 (23.5%)
	Missing	84 (2.2%)
Menopausal status	pre	1565 (41.7%)
	post	2189 (58.3%)
Treatment group	Control	1898 (50.6%)
	Interventional	1856 (49.4%)

### Definition of Molecular Tumor Subtypes

**TABLE F2:** Definition of molecular tumor subtypes in the SUCCESS‐A study data.

Subgroup	HER2	ER/PR	grade
HER2 positive	HER2+	any	any
Luminal A‐like	HER2−	ER+ and/or PR+	G1 or G2
Luminal B‐like	HER2−	ER+ and/or PR+	G3
Triple‐negative	HER2−	ER− and PR−	any

## References

[1] P. Royston and M. K. B. Parmar , “Restricted Mean Survival Time: An Alternative to the Hazard Ratio for the Design and Analysis of Randomized Trials With a Time‐To‐Event Outcome,” BMC Medical Research Methodology 13 (2013): 152. 24314264 10.1186/1471-2288-13-152 PMC3922847

[2] F. Ambrogi , S. Iacobelli , and P. K. Andersen , “Analyzing Differences Between Restricted Mean Survival Time Curves Using Pseudo‐Values,” BMC Medical Research Methodology 22 (2022): 71. 35300614 10.1186/s12874-022-01559-z PMC8931966

[3] Z. R. McCaw , A. R. Orkaby , L. J. Wei , D. H. Kim , and M. W. Rich , “Applying Evidence‐Based Medicine to Shared Decision Making: Value of Restricted Mean Survival Time,” American Journal of Medicine 132 (2019): 13–15. 30076822 10.1016/j.amjmed.2018.07.026

[4] P. Royston and M. K. B. Parmar , “The Use of Restricted Mean Survival Time to Estimate the Treatment Effect in Randomized Clinical Trials When the Proportional Hazards Assumption Is in Doubt,” Statistics in Medicine 30 (2011): 2409–2421. 21611958 10.1002/sim.4274

[5] A. Ni , Z. Lin , and B. Lu , “Stratified Restricted Mean Survival Time Model for Marginal Causal Effect in Observational Survival Data,” Annals of Epidemiology 64 (2021): 149–154. 34619324 10.1016/j.annepidem.2021.09.016 PMC8629851

[6] H. Uno , B. Claggett , L. Tian , et al., “Moving Beyond the Hazard Ratio in Quantifying the Between‐Group Difference in Survival Analysis,” Journal of Clinical Oncology 32 (2014): 2380–2385. 24982461 10.1200/JCO.2014.55.2208 PMC4105489

[7] H. M. Dehbi , P. Royston , and A. Hackshaw , “Life Expectancy Difference and Life Expectancy Ratio: Two Measures of Treatment Effects in Randomised Trials With Non‐Proportional Hazards,” British Medical Journal 357 (2017): 2250. 10.1136/bmj.j2250 PMC5444092 28546261

[8] M. J. Stensrud and M. A. Hernán , “Why Test for Proportional Hazards?,” Journal of the American Medical Association 323 (2020): 1401–1402. 32167523 10.1001/jama.2020.1267 PMC11983487

[9] P. Andersen , M. Hansen , and J. Klein , “Regression Analysis of Restricted Mean Survival Time Based on Pseudo‐Observations,” Lifetime Data Analysis 10 (2005): 335–350. 10.1007/s10985-004-4771-0 15690989

[10] L. Breiman , “Random Forests,” Machine Learning 45 (2001): 5–32.

[11] U. B. Mogensen and T. A. Gerds , “A Random Forest Approach for Competing Risks Based on Pseudo‐Values,” Statistics in Medicine 32 (2013): 3102–3114. 23508720 10.1002/sim.5775

[12] S. E. Leurgans , “Linear Models, Random Censoring and Synthetic Data,” Biometrika 74 (1987): 301–309.

[13] L. Tian , H. Fu , S. J. Ruberg , H. Uno , and L. J. Wei , “Efficiency of Two Sample Tests via the Restricted Mean Survival Time for Analyzing Event Time Observations,” Biometrics 74 (2018): 694–702. 28901017 10.1111/biom.12770 PMC5847424

[14] B. Huang and P. F. Kuan , “Comparison of the Restricted Mean Survival Time With the Hazard Ratio in Superiority Trials With a Time‐To‐Event End Point,” Pharmaceutical Statistics 17 (2018): 202–213. 29282880 10.1002/pst.1846

[15] R. Rong , J. Ning , and H. Zhu , “Regression Modeling of Restricted Mean Survival Time for Left‐Truncated Right‐Censored Data,” Statistics in Medicine 41 (2022): 3003–3021. 35708238 10.1002/sim.9399 PMC10014036

[16] L. Tian , L. Zhao , and L. J. Wei , “Predicting the Restricted Mean Event Time With the Subject's Baseline Covariates in Survival Analysis,” Biostatistics 15 (2014): 222–233. 24292992 10.1093/biostatistics/kxt050 PMC3944973

[17] X. Wang and D. Schaubel , “Modeling Restricted Mean Survival Time Under General Censoring Mechanisms,” Lifetime Data Analysis 24 (2018): 176–199. 28224260 10.1007/s10985-017-9391-6 PMC5565738

[18] T. Hasegawa , S. Misawa , S. Nakagawa , et al., “Restricted Mean Survival Time as a Summary Measure of Time‐To‐Event Outcome,” Pharmaceutical Statistics 19 (2020): 436–453. 32072769 10.1002/pst.2004

[19] D. H. Kim , H. Uno , and L. J. Wei , “Restricted Mean Survival Time as a Measure to Interpret Clinical Trial Results,” JAMA Cardiology 2 (2017): 1179–1180. 28877311 10.1001/jamacardio.2017.2922 PMC6359932

[20] P. K. Andersen and P. M. Pohar , “Pseudo‐Observations in Survival Analysis,” Statistical Methods in Medical Research 19 (2010): 71–99. 19654170 10.1177/0962280209105020

[21] F. Graw , T. A. Gerds , and M. Schumacher , “On Pseudo‐Values for Regression Analysis in Competing Risks Models,” Lifetime Data Analysis 15 (2009): 241–255. 19051013 10.1007/s10985-008-9107-z

[22] M. Overgaard , E. T. Parner , and J. Pedersen , “Asymptotic Theory of Generalized Estimating Equations Based on Jack‐Knife Pseudo‐Observations,” Annals of Statistics 45 (2017): 1988–2015.

[23] J. Robins , “A New Approach to Causal Inference in Mortality Studies With a Sustained Exposure Period ‐ Application to Control of the Healthy Worker Survivor Effect,” Mathematical Modelling 7 (1986): 1393–1512.

[24] J. M. Snowden , S. Rose , and K. M. Mortimer , “Implementation of g‐Computation on a Simulated Data Set: Demonstration of a Causal Inference Technique,” American Journal of Epidemiology 173 (2011): 731–738. 21415029 10.1093/aje/kwq472 PMC3105284

[25] C. Molnar , Interpretable Machine Learning ‐ A Guide for Making Black Box Models Explainable, 2nd ed. (Independently published, 2022).

[26] A. de Gregorio , L. Häberle , P. A. Fasching , et al., “Gemcitabine as Adjuvant Chemotherapy in Patients With High‐Risk Early Breast Cancer – Results From the Randomized Phase III SUCCESS‐A Trial,” Breast Cancer Research 22 (2020): 111. 33097092 10.1186/s13058-020-01348-w PMC7583247

[27] A. Schenk , M. Berger , and M. Schmid , “Pseudo‐Value Regression Trees,” Lifetime Data Analysis 30 (2024): 439–471. 38403840 10.1007/s10985-024-09618-x PMC11297840

[28] L. Breiman , J. Friedman , C. J. Stone , and R. A. Olshen , Classification and Regression Trees (Taylor & Francis, 1984).

[29] T. Hothorn , K. Hornik , and A. Zeileis , “Unbiased Recursive Partitioning: A Conditional Inference Framework,” Journal of Computational and Graphical Statistics 15 (2006): 651–674.

[30] B. M. Greenwell , Tree‐Based Methods for Statistical Learning in R (Chapman & Hall/CRC, 2022).

[31] R. de Bin , S. Janitza , W. Sauerbrei , and A. L. Boulesteix , “Subsampling Versus Bootstrapping in Resampling‐Based Model Selection for Multivariable Regression,” Biometrics 72 (2016): 272–280. 26288150 10.1111/biom.12381

[32] L. Hu , J. Ji , and F. Li , “Estimating Heterogeneous Survival Treatment Effect in Observational Data Using Machine Learning,” Statistics in Medicine 40 (2021): 4691–4713. 34114252 10.1002/sim.9090 PMC9827499

[33] R Core Team , R: A Language and Environment for Statistical Computing. R Foundation for Statistical Computing, version 4.1.2 (2021).

[34] T. M. Therneau and P. M. Grambsch , Modeling Survival Data: Extending the Cox Model (Springer, 2000).

[35] C. A. Hudis , W. E. Barlow , J. P. Costantino , et al., “Proposal for Standardized Definitions for Efficacy End Points in Adjuvant Breast Cancer Trials: The STEEP System,” Journal of Clinical Oncology 25 (2007): 2127–2132. 17513820 10.1200/JCO.2006.10.3523

[36] A. Cwiling , V. Perduca , and O. Bouaziz , “A Comprehensive Framework for Evaluating Time to Event Predictions Using the Restricted Mean Survival Time,” arXiv: arXiv.2306.16075.

[37] A. S. Coates , E. P. Winer , A. Goldhirsch , et al., “Tailoring Therapies – Improving the Management of Early Breast Cancer: St.Gallen International Expert Consensus on the Primary Therapy of Early Breast Cancer 2015,” Annals of Oncology 26 (2015): 1533–1546. 25939896 10.1093/annonc/mdv221 PMC4511219

[38] E. Senkus , S. Kyriakides , S. Ohno , et al., “Primary Breast Cancer: ESMO Clinical Practice Guidelines for Diagnosis, Treatment and Follow‐Up,” Annals of Oncology 26, no. Suppl. 5 (2015): v8–v30. 26314782 10.1093/annonc/mdv298

[39] C. M. Perou , T. Sørlie , M. B. Eisen , et al., “Molecular Portraits of Human Breast Tumours,” Nature 406 (2000): 747–752. 10963602 10.1038/35021093

[40] G. von Minckwitz , M. Untch , J. U. Blohmer , et al., “Definition and Impact of Pathologic Complete Response on Prognosis After Neoadjuvant Chemotherapy in Various Intrinsic Breast Cancer Subtypes,” Journal of Clinical Oncology 30 (2012): 1796–1804. 22508812 10.1200/JCO.2011.38.8595

[41] A. Goldhirsch , W. C. Wood , R. D. Gelber , A. S. Coates , B. Thürlimann , and H. J. Senn , “Meeting Highlights: Updated International Expert Consensus on the Primary Therapy of Early Breast Cancer,” Journal of Clinical Oncology 21 (2003): 3357–3365. 12847142 10.1200/JCO.2003.04.576

[42] M. A. Hernán , “The Hazards of Hazard Ratios,” Epidemiology 21 (2010): 13–15. 20010207 10.1097/EDE.0b013e3181c1ea43 PMC3653612

[43] V. Didelez and M. J. Stensrud , “On the Logic of Collapsibility for Causal Effect Measures,” Biometrical Journal 64 (2022): 235–242. 33576019 10.1002/bimj.202000305

[44] P. Y. Chen and A. A. Tsiatis , “Causal Inference on the Difference of the Restricted Mean Lifetime Between Two Groups,” Biometrics 57 (2001): 1030–1038. 11764241 10.1111/j.0006-341x.2001.01030.x

[45] M. A. Hernán and J. M. Robins , Causal Inference: What if (CRC Press, 2024).

[46] L. Tian , H. Jin , H. Uno , et al., “On the Empirical Choice of the Time Window for Restricted Mean Survival Time,” Biometrics 76 (2020): 1157–1166. 32061098 10.1111/biom.13237 PMC8687138

[47] Y. Zhong and D. E. Schaubel , “Restricted Mean Survival Time as a Function of Restriction Time,” Biometrics 78 (2022): 192–201. 33616953 10.1111/biom.13414 PMC8184877

[48] T. Coleman , L. Mentch , D. Fink , et al., “Statistical Inference on Tree Swallow Migrations With Random Forests,” Journal of the Royal Statistical Society: Series C: Applied Statistics 69 (2020): 973–989.

[49] M. N. Wright and A. Ziegler , “Ranger: A Fast Implementation of Random Forests for High Dimensional Data in C++ and R,” Journal of Statistical Software 77 (2017): 1–17.

[50] L. Zhao and D. Feng , “Deep Neural Networks for Survival Analysis Using Pseudo Values,” IEEE Journal of Biomedical and Health Informatics 24 (2020): 3308–3314. 32167918 10.1109/JBHI.2020.2980204 PMC8056290

[51] L. Zhao , “Deep Neural Networks for Predicting Restricted Mean Survival Times,” Bioinformatics 36 (2021): 5672–5677. 33399818 10.1093/bioinformatics/btaa1082 PMC8023687

[52] K. Goldfeld and J. Wujciak‐Jens , “simstudy: Illuminating Research Methods Through Data Generation,” R package version 0.7.1, (2023).

[53] M. Pohar Perme and M. Gerster , pseudo: Computes Pseudo‐Observations for Modeling, R package version 1.4.3 (2017).

[54] T. Hothorn , H. Seibold , and A. Zeileis , “partykit: A toolkit for recursive partytioning,” R package version 1.2.20 (2023).

[55] T. M. Therneau , “survival: A package for survival analysis in R,” R package version 3.5.7 (2023).

[56] S. Højsgaard , U. Halekoh , and J. Yan , “The R Package Geepack for Generalized Estimating Equations,” Journal of Statistical Software 15 (2006): 1–11.

[57] C. Molnar , B. Bischl , and G. Casalicchio , “Iml: An R Package for Interpretable Machine Learning,” Journal of Open Source Software 3 (2018): 786.

